# Sustained release of ubiquitin-like protein ISG-15 enhances tendon-to-bone healing following anterior cruciate ligament reconstruction in a mouse model

**DOI:** 10.3389/fbioe.2025.1550584

**Published:** 2025-03-12

**Authors:** Jun-Cheng Yao, Jie-Xin Zhang, Xuan Wang, Yu-Hao Wu, Hao-Lin Ke, Jia-Rong Liang, Yan Shao, Jin-Tao Li, Yuan Liu, Dao-Zhang Cai, Jian-Ying Pan

**Affiliations:** ^1^ Department of Joint Surgery, Center for Orthopaedic Surgery, The Third Affiliated Hospital of Southern Medical University, Guangzhou, China; ^2^ Department of Orthopedics, Orthopedic Hospital of Guangdong Province, Academy of Orthopedics·Guangdong Province, The Third Affiliated Hospital of Southern Medical University, Guangzhou, China; ^3^ The Third School of Clinical Medicine, Southern Medical University, Guangzhou, China; ^4^ Guangdong Provincial Key Laboratory of Bone and Joint Degeneration Diseases, Guangzhou, China; ^5^ Department of Orthopedics, Inner Mongolia Autonomous Region Hospital of Traditional Chinese Medicine, Hohhot, China

**Keywords:** tendon-bone healing, ISG15, osteogenic differentiation, anterior cruciate ligament reconstruction, sustain release

## Abstract

The process of tendon-to-bone healing is regulated by several proteins and cytokines that play critical roles in shaping biomechanical properties and functional recovery. Among these, the ubiquitin-like protein ISG-15 has been reported to have a beneficial effect on tissue repair. However, its specific function in tendon-to-bone interface regeneration has not been well characterized. This study investigated the function of ISG15 *in vitro* and addressed its *in vivo* effects on tendon and bone healing. In this study, wild-type C57/BL6 mice underwent anterior cruciate ligament (ACL) reconstruction surgery, with a sustained-release hydrogel containing ISG15 protein injected into the bone tunnels in the treatment group. To assess its therapeutic potential, bone-tendon interface growth was evaluated through histological staining, while micro-computed tomography (Micro-CT) was employed to quantify newly formed bone and bone density within the bone tunnels. Additionally, biomechanical testing was performed to measure the mechanical strength of the grafted tendons, and immunohistochemistry was conducted to detect the expression of Runx2 and osteocalcin (OCN) at the bone-tendon interface. *In vitro* results showed that an appropriate concentration of ISG-15 has the ability to promote osteogenic differentiation of bone marrow mesenchymal stem cells. Also, In the *in vivo* experiments, the local application of ISG15 protein significantly reduced inflammatory tissue growth during the early stages of healing and minimized bone resorption in the later stages. Furthermore, Micro-CT analysis showed an increased volume of newly formed bone in the treatment group, while biomechanical testing demonstrated enhanced mechanical strength of the grafted tendons. In summary, this study suggests that the localized sustained release of ISG15 protein during ACL reconstruction facilitates tendon-to-bone interface repair by promoting bone ingrowth, ultimately leading to improved biomechanical properties and functional recovery.

## Introduction

Anterior cruciate ligament (ACL) rupture is a prevalent sports injury. Untreated ACL dysfunction can severely impair knee motor function, lead to early-onset knee osteoarthritis, reduce quality of life, and in severe cases, cause joint disability (Sanders et al., 2017; Widner et al., 2019). Surgical reconstruction of the ACL is the standard treatment to restore knee function. Stabilizing the bone-tendon interface enables patients to regain their athletic abilities (Atesok et al., 2014). Recent European data indicates approximately 165,000 ACL reconstruction (ACLR) surgeries are performed annually (Longo et al., 2021). However, 3%–7% of patients require revision surger (Wolfson et al., 2023). In the United States, over 10,000 patients undergo secondary ACL reconstruction each year (Lyman et al., 2009).

Despite successful surgical techniques, the repaired tendon-bone interface often struggles to fully regain its anatomical and biomechanical function. Previous studies have confirmed that successful ACL reconstruction relies on adequate osteointegration between the tendon graft and the bone tunnel (Rodeo et al., 2006; Packer et al., 2014). A significant challenge is the early, robust inflammatory response, which can lead to scar formation at the tendon-bone interface (Wang et al., 2019). This weakens the tendon graft and increases the risk of re-tearing. Furthermore, scar tissue interferes with osteogenesis and bone ingrowth (Higuchi et al., 2006), resulting in poor mechanical properties that can lead to graft dislocation or failure, ultimately impairing knee joint stability. Immediately after reconstruction surgery, numerous immune cells are rapidly recruited to the reconstructed tendon interface, initiating an inflammatory process at the tendon-to-bone interface (Lebaschi et al., 2018; Kamalitdinov et al., 2020). During inflammation, macrophages differentiate into M1 and M2 phenotypes. After ACLR, monocyte-derived macrophages can polarize into M1 macrophages, characterized by their pro-inflammatory function (Kawamura et al., 2005). At the micro level, pro-inflammatory factors accumulate in the surrounding tendon tissue during the inflammatory stage of post-surgical healing, leading to collagen disarray, myotendinous degeneration, and impeded tendon neovascularization (Higuchi et al., 2006). Subsequently, M1 macrophages are gradually replaced by M2 macrophages, which exhibit anti-inflammatory and pro-healing functions (Sindrilaru et al., 2011) through the secretion of cytokines.

Cytokines play a pivotal role in this intricate healing process (Bosurgi et al., 2017; Shook et al., 2018). Beyond modulating the inflammatory response, these cytokines can also influence new bone ingrowth. Studies have demonstrated that successful tendon-bone healing depends on new bone ingrowth, mineralization, and maturation around the bone tunnel (Bedi et al., 2010; Brophy et al., 2011; Lu et al., 2020). Consequently, researchers have explored various strategies to enhance postoperative bone formation. Among these, the application of cytokines has garnered significant attention, and the search for suitable cytokines to promote tendon-bone healing has become an increasingly important focus in tissue engineering and biomaterial synthesis (Tian et al., 2023).

In their search for key factors, Fujii T et al. used scRNA-seq to identify a subset of CX3CR1+ CCR2+ cells at the tendon-bone interface following ACLR. These cells exhibited a pronounced interferon (IFN) response, characterized by the upregulation of interferon-stimulated genes (ISGs) (Fujii et al., 2022). Although the role of the IFN response in tendon-bone healing is not fully understood, it has been linked to tissue repair, collagen synthesis, fibroblast proliferation, and bone formation and remodeling (Kim et al., 2003; Guiducci et al., 2010; Ivashkiv, 2018). Previous studies have identified ISG15, a ubiquitin-like protein, as one of the most rapidly induced genes among ISGs, with a variety of biological functions (Perng and Lenschow, 2018). However, its specific role in tendon bone healing remains unknown.

To investigate the role of ISG15 in tendon-to-bone healing, we developed a sustained-release rISG15 hydrogel and evaluated its release profile both *in vitro* and *in vivo*. Using a mouse ACLR model, we injected the hydrogel at the tendon-bone interface to achieve sustained rISG15 release. This delivery method promoted tendon-to-bone healing by enhancing osteogenic differentiation at the interface. Therefore, our study suggests a promising therapeutic strategy for improving tendon-to-bone healing outcomes.

## Method and materials

### Animal study design and surgical procedure

**FIGURE 1:**
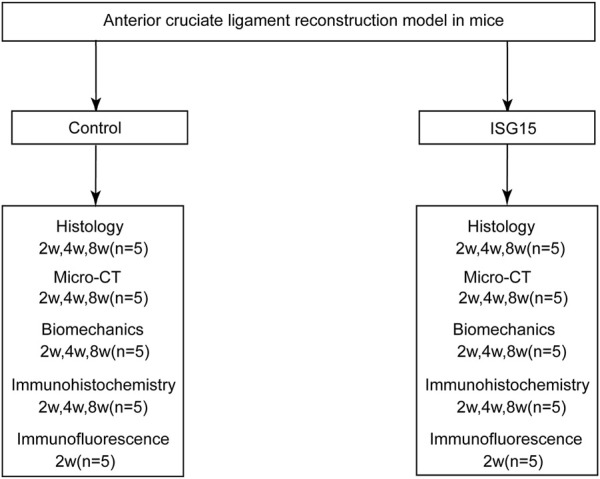
Experimental design diagram.

**FIGURE 2:**
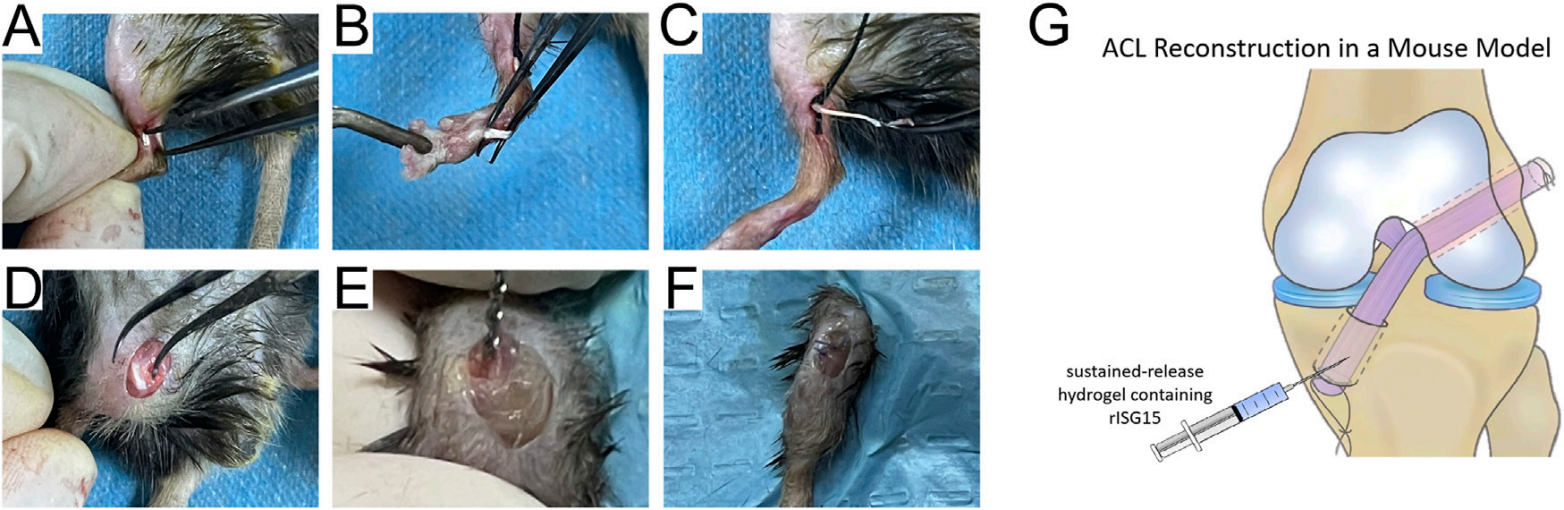
Schematic of the operations on mice. **(A–F)** Surgical procedure for establishing the ACLR model in mice. **(G)** The graphic illustration of the hydrogel injection into the bone canal.

All animal care and experimental procedures were approved by the Laboratory Animal Ethics Committee of Southern Medical University. According to the experimental design (Figure 1), 8-week-old mice were housed in a specific pathogen-free facility under controlled conditions (23°C, 40% humidity). The ACLR model (Figure 2G) was constructed as previously described (Yu et al., 2020). Anesthesia was administered via intraperitoneal injection of pentobarbital sodium (50 mg/kg), with the depth verified by the absence of pedal reflex and response to external stimuli. After the experiment, the treated mice were euthanized via cervical dislocation under deep anesthesia induced with the same dose of pentobarbital sodium, in compliance with the institutional and international ethical guidelines. Under anesthesia, the flexor digitorum longus tendon was aseptically harvested and preserved in 4°C micro-hydrogel (without or with rISG15) for graft preparation (Figures 2A–C). Subsequently, the joint cavity was exposed, the native ACL was transected, and tibial and femoral bone tunnels of approximately 0.6-mm diameter were drilled (Figures 2D, E). A 10-μL volume of micro-hydrogel, without or with rISG15, was then injected into the bone tunnels, and the tendon graft was subsequently guided into the tunnels with a guidewire and sutures, with both the ends fixed securely. Prior to suturing, the hydrogel was injected between the graft and the bone tunnel using a microinjector (Hamilton 600 series, China), allowing the space between the bone tunnel and the graft to be filled with hydrogel. The joint capsule and skin were then sutured meticulously in layers (Figure 2F), and the animals were allowed unrestricted cage activity after the surgery. At 2, 4, and 8 weeks after the surgery, knee joint samples were collected for immunofluorescence and immunohistochemistry so as to evaluate the protein expression, histological staining to assess tissue morphology, micro-CT for structural analysis, and biomechanical testing to measure the graft strength.

### Formulation of a protein slow-release hydrogel containing ISG15

The sustained-release hydrogel was prepared using previously reported methods (Chen et al., 2021; Wu et al., 2022). Briefly, fibrinogen and thrombin (Sigma-Aldrich, United States of America) were added to the thrombin solution in accordance with the manufacturer’s instructions. A mixture of 200 µL fibrinogen and 40 µL of the thrombin solution was prepared using concentrations consistent with those suggested in previous studies to create the fibrin sealant. rISG15 protein (LifeSpan BioSciences, United States of America) was then added to the thrombin solution. For the control group, fibrin sealants were prepared similarly, without the addition of rISG15 protein.

### Biocompatibility of sustained-release hydrogel *in vitro*

To assess the *in vitro* biocompatibility of the sustained-release hydrogel, live/dead cell staining was performed. Femurs and tibiae were isolated from 4-week-old mice, and the bone marrow was flushed out, filtered, and plated into culture dishes for 48 h. Non-adherent cells were then removed, and adherent cells were cultured in α-MEM supplemented with 10% FBS and antibiotics (100 U/mL penicillin, 100 μg/mL streptomycin) at 37°C, 95% humidity, and 5% CO_2_. A 1.0 g hydrogel sample was degraded in 10 mL of 1 M NaOH to yield a 1× degradation product solution, which was then neutralized to pH 7.4 and sterilized via filtration through a 0.22-μm Teflon filter. In a 96-well plate, at least three repetitive testing wells were prepared for both the test and control groups. Each well was seeded with 100 μL of the bone marrow-derived mesenchymal stem cell (BMSC) suspension (5 × 10^4^ cells/mL) in complete α-MEM, followed by 24 h of incubation, after which 10 μL of the degradation product was added to the test group wells. The morphology and viability of the cells were observed using a Live/Dead Viability/Cytotoxicity Kit and an inverted fluorescence microscope (Leica DMI4000 B). This experimental setup assessed the cell viability and morphology following exposure to hydrogel degradation products, providing information on the biocompatibility of the hydrogel. The result of Live/Dead Staining are presented in Supplementary Figure S1.

### ELISA

The experimental cells were cultured in six-well plates, and 200 μL of hydrogel, without or with rISG15, was added to each well. The plates were incubated at 37°C with 5% CO_2_ in a humidified environment. At specific time points, the supernatant was collected, and the rISG15 release was quantified using the ISG15 ELISA kit (Signalway Antibody, United States of America) according to the manufacturer’s instructions. Next, absorbance at 450 nm was measured, and the rISG15 concentration in the hydrogel was determined with reference to a standard curve.

### CCK-8 assay

To determine the impact of recombinant murine ISG15 (rISG15) on cell viability, we conducted a Cell Counting Kit-8 (CCK-8) assay using BMSCs isolated from mice. BMSCs were seeded in 96-well plates at a density of 5 × 10^3^ cells per well and cultured under standard conditions (37°C, 5% CO_2_). Following cell adhesion, the BMSCs were treated with rISG15 at concentrations of 5, 10, 20, 50, and 100 ng/mL for 24, 48, and 72 h. Cell viability was then assessed using the CCK-8 assay kit (Solarbio, China), adhering strictly to the manufacturer’s protocol. At each designated time point, 10 μL of CCK-8 solution was added to each well, followed by a 2-h incubation period at 37°C. Absorbance was measured at 450 nm using a microplate reader. All experiments were performed in triplicate, and the resulting data are presented as mean ± standard deviation (SD). Statistical significance was determined via one-way ANOVA, with a threshold of p < 0.05 defining significance.

### Induction of osteogenic and chondrogenic differentiation

BMSCs were isolated following a previously established protocol and employed for subsequent induction experiments. Osteogenic or chondrogenic differentiation was initiated by culturing the cells for 14 days in either osteogenic or chondrogenic differentiation medium (Pythonbio, China). As a control, one group of cells was cultured without the addition of rISG15 protein, while the experimental group was cultured in the presence of rISG15 (20 ng/mL). On day 14, osteogenic differentiation in both groups was assessed via Alizarin Red S staining (Solarbio, China). Calcium deposition, indicative of osteogenic differentiation, was quantified by measuring absorbance at 560 nm. Furthermore, the expression of *Isg15* during osteogenic (or chondrogenic) differentiation of BMSCs was evaluated by quantitative real-time PCR (qRT-PCR).

### Quantitative RT-PCR (qRT-PCR)

Following a 14-day culture period under osteogenic or chondrogenic differentiation conditions, with or without rISG15 supplementation, total RNA was extracted from the BMSCs using FreeZol Reagent (Vazyme, China). qRT-PCR was then performed utilizing SYBR Green Fast qPCR Mix (RK21203, ABclonal, China) on the LightCycler^®^ 96 Real-Time PCR System (Roche, United States of America). The 2^-ΔΔCt method was employed to analyze relative gene expression levels and thus evaluate the impact of rISG15 on osteogenic and chondrogenic differentiation. The specific primer sequences utilized in this analysis are detailed in Table 1.

**TABLE 1:** Primer sequences used for qRT-PCR.

Gene	Direction	Primer sequence (5′–3′)
Mouse GAPDH	Forward	AAA TGG TGA AGG TCG GTG TGA AC
Mouse GAPDH	Reverse	CAA CAA TCT CCA CTT TGC CAC TG
Mouse Runx2	Forward	TCC CCG GGA ACC AAG AAG GCA
Mouse Runx2	Reverse	AGG GAG GGC CGT GGG TTC TG
Mouse OCN	Forward	CTG ACC TCA CAG ATC CCA AGC
Mouse OCN	Reverse	TGG TCT GAT AGC TCG TCA CAA G
Mouse Collagen Ⅱ	Forward	CAC CCT CAA ATC CCT CAA CAA TCA G
Mouse Collagen Ⅱ	Reverse	TGT CTT TCG TCT TGC TGG TCC ACC
Mouse Sox9	Forward	TAC CTA CGG CAT CAG CAG CTC
Mouse Sox9	Reverse	TTG CCT TCA CGT GGC TTT AAG

### Micro-CT analysis

Knee joints were harvested from mice at 2, 4, and 8 weeks post-ACLR surgery, with five specimens per time point selected for analysis. The femoral tunnel microstructure was then examined, and three-dimensional image reconstruction and morphometric analysis were performed using the acquired data and dedicated image analysis software. Scanning parameters were optimized as follows: 15 μm layer thickness, 55 kVp voltage, 145 μA current, and 8 W power. An initial pre-scan of the entire sample was conducted, followed by a high-resolution scan focused on the region of interest (Figure 5A). The resulting scanned images were processed using image analysis software to delineate the segment extending from the femoral tunnel entrance to the articular cavity, thereby enabling the calculation of bone volume fraction (BV/TV) and bone mineral density (BMD).

### Biomechanical testing

At 2, 4, and 8 weeks post-surgery, five mice per time point were selected for analysis. The distal tibia and proximal femur were carefully dissected, and the surrounding soft tissues and ligaments of the knee joint were meticulously excised, preserving only the reconstructed ACL. Each specimen was then encased in cushioning cotton to minimize mechanical interference and securely affixed within the testing apparatus (Figure 5B). To mitigate mechanical artifacts and ensure the fidelity of biomechanical testing, the suture knots at both ends of the reconstructed ligament were removed. Subsequently, the ultimate tensile load was determined using a high-precision mechanical testing system (Electroforce 3,200, TABOSE, United States of America) by preloading the graft to zero force and applying a displacement rate of 0.5 mm/s until either graft pullout from the bone tunnel or ligament rupture occurred. A load-displacement curve was generated, and the slope of the initial linear region approaching the curve’s maximum was calculated to quantify graft stiffness. To minimize inter-specimen variability, graft dimensions were standardized.

### Histology staining

At 2, 4, and 8 weeks post-ACLR, five mice per group were euthanized. Knee joints were then harvested, fixed in 4% formaldehyde, decalcified in EDTA, dehydrated, and embedded in paraffin. Serial sections of 3-μm thickness were prepared along the sagittal plane of each knee joint and subsequently stained with hematoxylin and eosin (H&E), Masson’s trichrome, and Safranin O-fast green. Histological scoring was performed according to the method described by Zhao et al. (2024). For evaluating tendon-bone interface healing histologically, a semi-quantitative scoring system was implemented, encompassing five parameters: separation, cellularity, extent of fibrocartilage tissue surrounding the tendon, interface tissue transition from bone to tendon, and tidemark presence. Separation was scored on a scale of 0–4, where 0 indicated complete separation characterized exclusively by fibrovascular tissue, and four represented minimal separation with fibrocartilage containing mature chondrocytes covering ≥50% of the interface. Cellularity, reflecting the cellular morphology of the interface tissue, was scored from 0 for fibrovascular tissue alone to four for fibrocartilage containing mature chondrocytes covering ≥50% of the area. The extent of fibrocartilage surrounding the tendon was scored from 0, denoting the absence of visible fibrocartilage, to 4, denoting that ≥75% of the tendon perimeter was surrounded by fibrocartilage. The interface transition from bone to tendon was evaluated on a scale of 0–4, with 0 indicating a discontinuous transition and 4 representing a predominantly distinct and continuous transition spanning ≥75% of the interface. Finally, tidemark visibility was scored from 0 for an absent tidemark to4 for a tidemark extending across ≥75% of the interface. Two blinded observers independently assessed the slides, and any scoring discrepancies were resolved through consensus discussion.

### Immunofluorescence and immunohistochemical staining

Knee joint specimens from mice at 2, 4, and 8 weeks of age were meticulously processed for immunofluorescence and immunohistochemical staining. Following a 1-month decalcification in EDTA solution, the specimens were embedded in paraffin and sectioned into 4 μm slices. The sections were then heated in an oven at 65°C for 1 h to facilitate deparaffinization with xylene, followed by rehydration through a graded series of ethanol. Antigen retrieval was achieved by heating the sections in sodium citrate buffer (pH 6.0) at 65°C for 12 h. Afterward, the sections were incubated in hydrogen peroxide solution for 15 min to quench endogenous peroxidase activity and subsequently blocked with 3% goat serum at room temperature for 1 h. Primary antibodies were applied and allowed to incubate overnight at 4°C, followed by a 1-h incubation with secondary antibodies at room temperature.

For immunohistochemical analysis, sections were developed using diaminobenzidine (DAB) (abs9211, Absin, China) as the chromogen, counterstained with hematoxylin, and rinsed with phosphate-buffered saline (PBS) for bluing. In the case of immunofluorescence, sections were incubated with fluorophore-conjugated secondary antibodies at room temperature for 1 h, followed by nuclear counterstaining with 4′,6-diamidino-2-phenylindole (DAPI). Images were then acquired using a confocal laser scanning microscope (FV3000, Olympus). The primary antibodies employed were as follows: Runx2 (ab76956, Abcam, United Kingdom), osteocalcin (OCN; sc-390877, Santa Cruz, United States of America), and ISG15 (sc-166755, Santa Cruz). The secondary antibodies utilized included Alexa Fluor^®^ 594-conjugated Goat Anti-Mouse IgG (ab150116, Abcam, United Kingdom) and Goat Anti-Mouse IgG H&L (HRP) (ab6789, Abcam, United Kingdom).

### Statistical analysis

Statistical analyses were performed using SPSS software (version 26.0, IBM, United States of America), and data are presented as mean ± SD. Data visualization was achieved using GraphPad Prism software (version 9, San Diego, CA, United States of America). For quantitative comparisons among multiple groups, a one-way ANOVA was conducted, followed by Tukey’s post hoc test for pairwise comparisons. Student’s t-test was employed for pairwise comparisons between the two groups. Statistical significance was defined as *p* < 0.05 for all analyses, with significance denoted as *(*p* < 0.05), **(*p* < 0.01), and ***(*p* < 0.001).

## Result

### Sustained-release characteristics of rISG15-Loaded hydrogel

**FIGURE 3:**
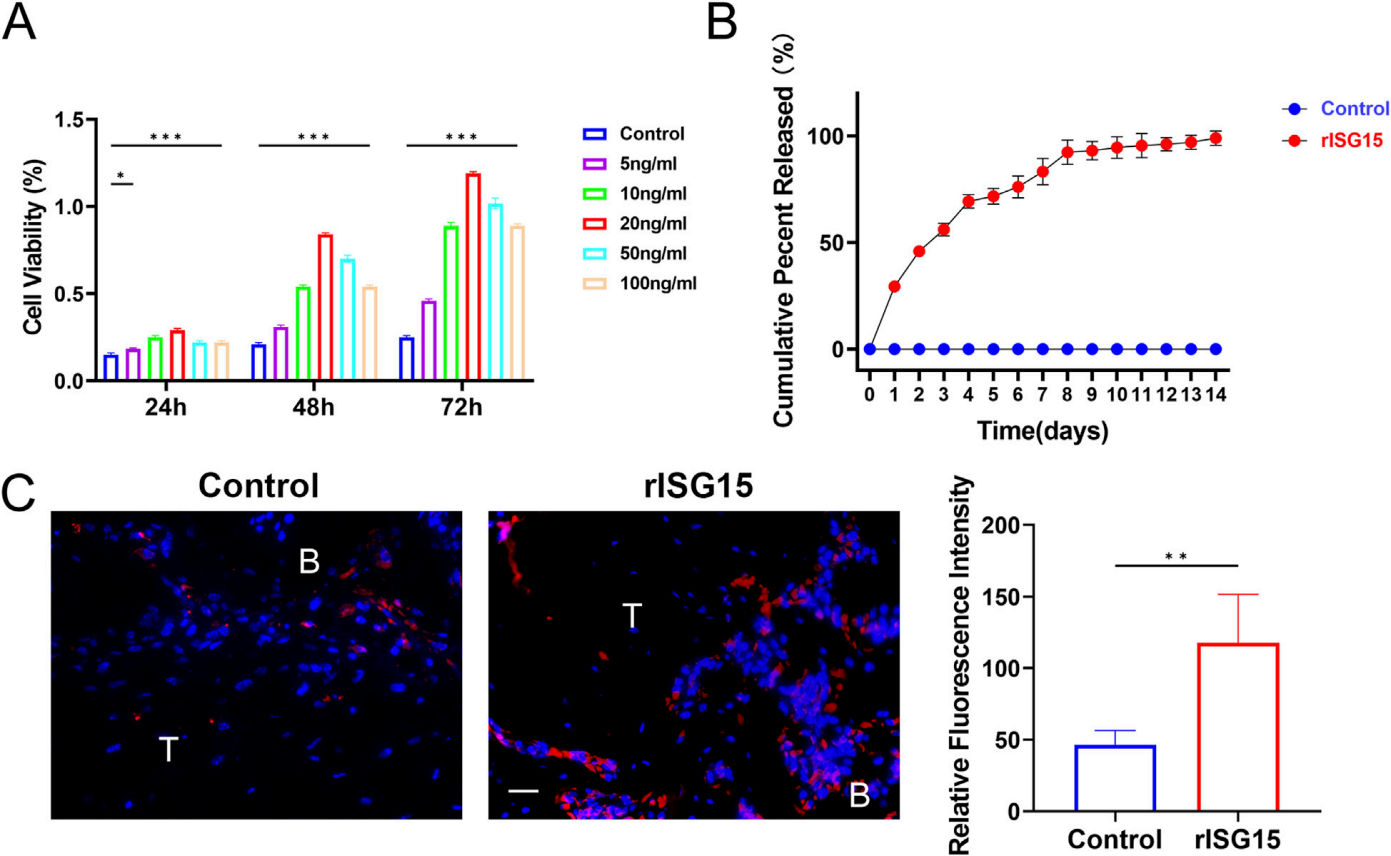
The sustained-release properties of the rISG15 hydrogel *in vitro* and *in vivo*. **(A)** Quantitative analysis of the CCK-8 assay demonstrates the effects of different concentrations of rISG15 on the viability of BMSCs (n = 3 per group). **(B)** Quantitative analysis of ELISA results illustrates the sustained-release performance of the rISG15 hydrogel at various time points (n = 3 per time point). **(C)** Immunofluorescence staining and quantitative analysis reveal the sustained release of rISG15 at the tendon-bone interface in mice at the 2-week time point (n = 5 per group, scale bar = 20 μm).

The hydrogel incorporating recombinant ISG15 (rISG15) demonstrated effective sustained-release characteristics under physiological conditions. Specifically, the hydrogel facilitated controlled and prolonged release of rISG15 at the tendon-bone interface in mice, ensuring sustained stimulation within the interfacial milieu. BMSCs isolated from mice were subjected to *in vitro* stimulation with recombinant rISG15 at concentrations of 5, 10, 20, 50, and 100 ng/mL for 24, 48, and 72 h, with the control group receiving an equivalent volume of PBS. The CCK-8 assay revealed that rISG15 at 20 ng/mL significantly augmented BMSC viability, exhibiting maximal effects across all time points examined (Figure 3A). Complementary *in vitro* release studies demonstrated a steady and gradual release of rISG15 from the hydrogel over a 2-week period in PBS at 37°C. ELISA further confirmed the hydrogel’s capacity for stable and prolonged delivery of recombinant rISG15 (Figure 3B). *In vivo* analyses utilizing a murine ACLR model demonstrated substantial deposition of hydrogel-delivered rISG15 at the tendon-bone interface after 2 weeks, a finding corroborated by immunofluorescence staining (Figure 3C). Quantitative assessment indicated that the relative fluorescence intensity of rISG15 in the hydrogel-treated group was significantly elevated compared to the control group (*P* < 0.05).

### Histological evaluation of the effect of rISG15-Loaded hydrogel on tendon-to-bone healing

**FIGURE 4:**
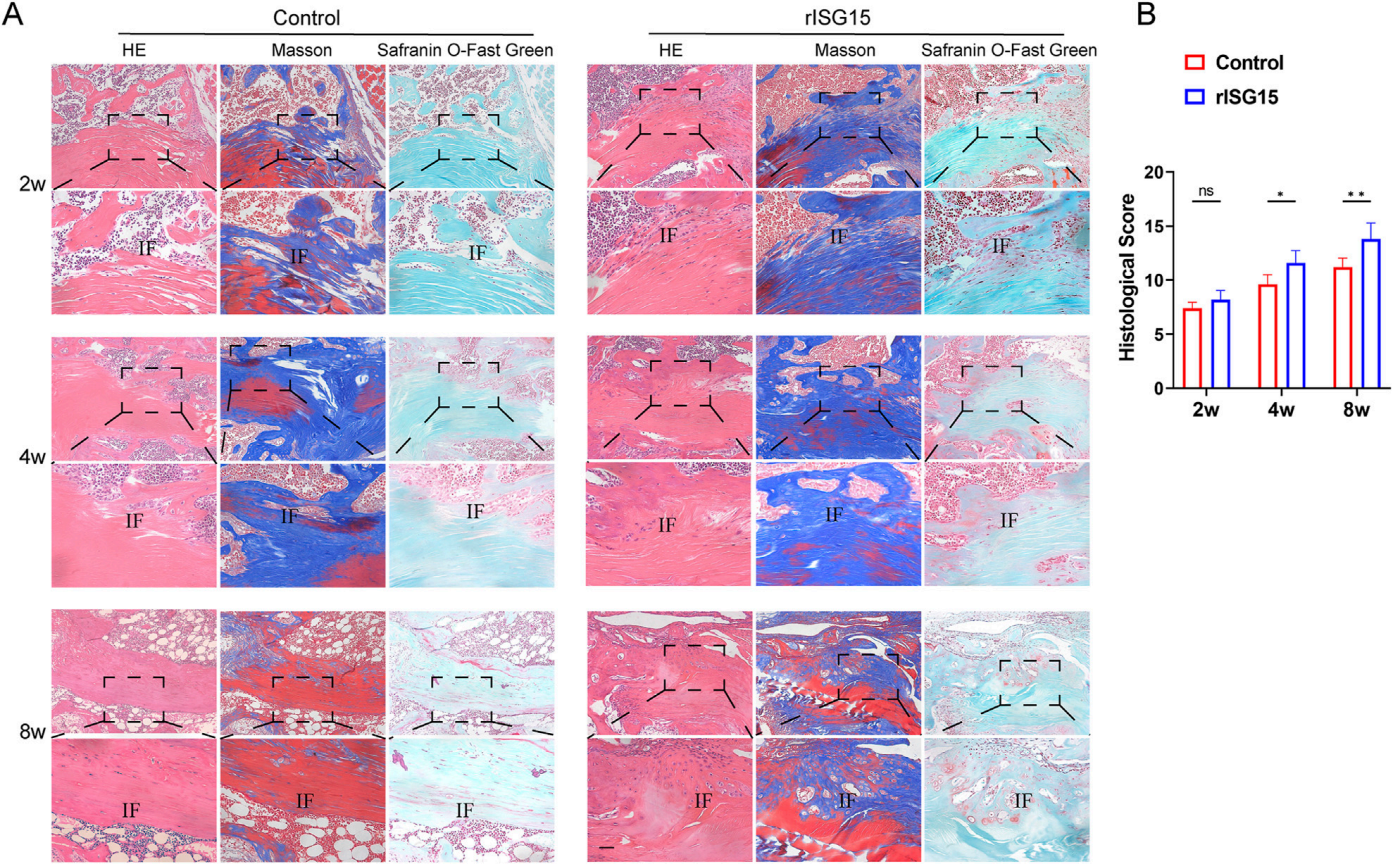
Histological staining reveals the effects of sustained-release rISG15 on tendon-bone healing at different time points. **(A, B)** Histological staining and quantitative analysis using a scoring system demonstrate the effects of sustained-release rISG15 on tendon-bone healing in mice at 2, 4, and 8 weeks (n = 5 per group, scale bar = 50 μm).

To ascertain the impact of rISG15-loaded hydrogel on tendon-bone healing, histological staining was performed at each designated time point (Figure 4A). At 2 weeks, H&E and Masson’s trichrome staining revealed a tenuous connection and sparse collagen fibers at the tendon-bone interface within the control group. Conversely, the rISG15-treated group exhibited more robust tendon-bone connections with denser collagen fibril deposition. Safranin O staining indicated limited fibrocartilage genesis in the control group, while the rISG15-treated group displayed nascent fibrocartilage development at the tendon-bone interface. At 4 weeks, H&E and Masson’s trichrome staining revealed irregular collagen fibril alignment and diminished staining intensity at the tendon-bone interface in the control group. The rISG15-treated group, however, demonstrated enhanced collagen deposition and a more consolidated interfacial structure. Safranin O staining further substantiated augmented fibrocartilage formation in the rISG15 group, with more intense cartilage matrix staining apparent at the interface. By 8 weeks, the rISG15-treated group exhibited a more organized tendon-bone interface, characterized by mature fibrocartilage and well-aligned collagen fibrils, a finding validated by all three staining methods. In contrast, the control group displayed incomplete fibrocartilage maturation, disorganized collagen architecture, and a lack of integrated tendon-bone continuity.

To quantify the observed histological disparities, histological scores were evaluated at 2, 4, and 8 weeks post-treatment (Figure 4B). At the 2-week time point, no statistically significant difference in histological scores was discernible between the control and rISG15-treated groups. However, by 4 and 8 weeks, the histological scores within the rISG15 group were significantly elevated relative to those in the control group (p < 0.05 and p < 0.01, respectively).

### Effects of sustained-release rISG15 on tunnel bone growth and graft mechanics via Micro-CT and biomechanics

**FIGURE 5:**
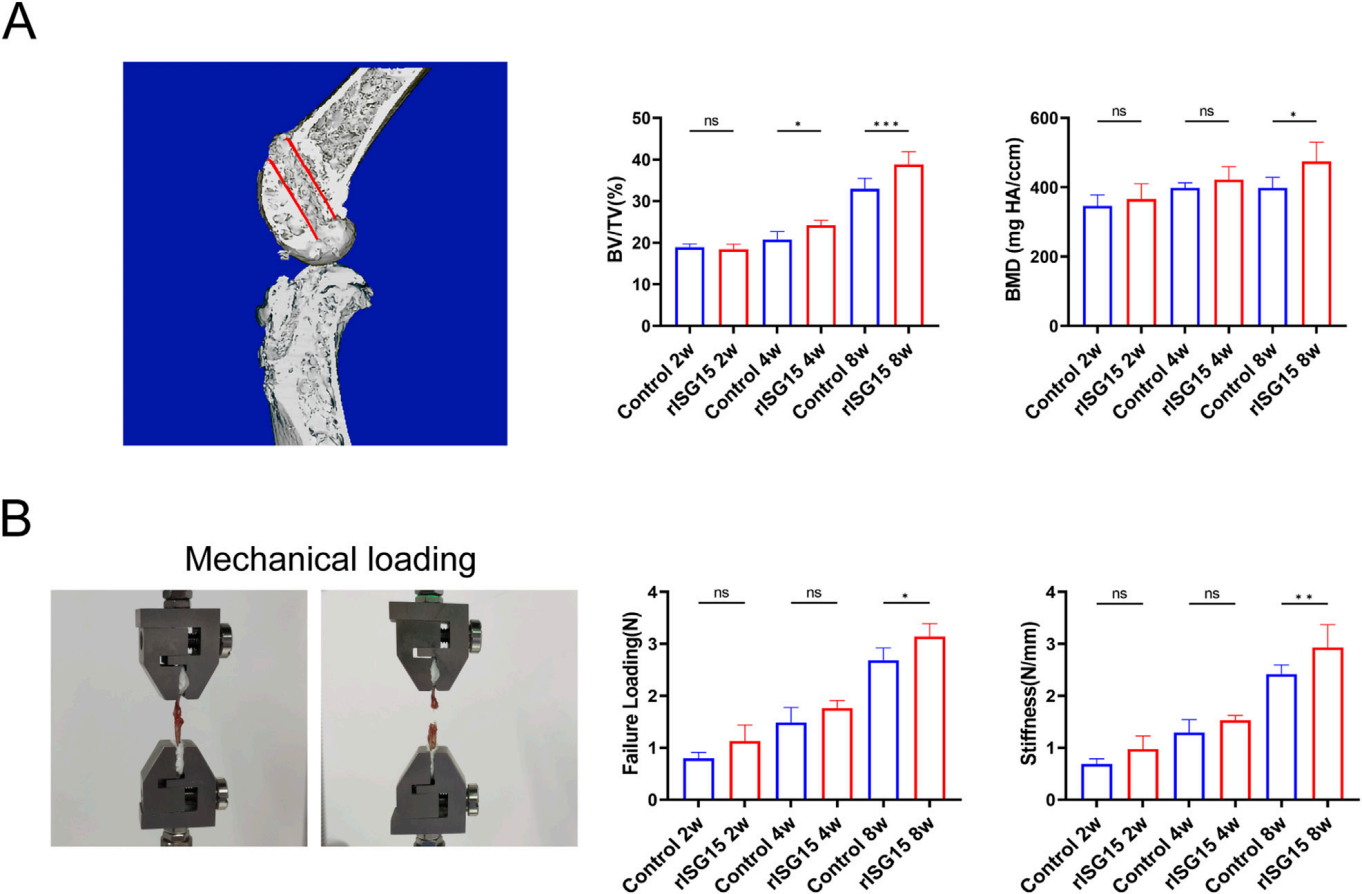
Micro-CT analysis and biomechanical testing demonstrate the effects of sustained-release rISG15 on tendon-bone healing. **(A)** In the 3D reconstruction, the red-marked regions indicate the measurement sites for new bone volume (BV/TV) and bone mineral density (BMD) within the bone tunnel of mice, with quantitative analysis performed (n = 5 per group). **(B)** Quantitative analysis shows the maximum load strength and graft stiffness of the reconstructed grafts in mice at different time points (n = 5 per group).

To assess the influence of sustained-release rISG15 hydrogel on bone tunnel regeneration and graft biomechanical integrity, micro-computed tomography (micro-CT) and biomechanical testing were conducted on specimens harvested at each time point. Micro-CT analysis revealed marked enhancements in bone formation within the rISG15-treated group compared to the control group (Figure 5A). At the 2-week interval, no significant differences in bone volume fraction (BV/TV) were observed between the groups. However, by week 4, the BV/TV in the rISG15-treated group was significantly elevated relative to the control group (p < 0.05). At week 8, the BV/TV exhibited a further increase in the rISG15 group (p < 0.001), indicative of robust and sustained osseous regeneration. Analogously, BMD values showed no significant disparities between the groups at 2 and 4 weeks. However, at 8 weeks, the BMD within the rISG15-treated group was significantly greater than that of the control group (p < 0.05). These observations underscore the capacity of the sustained-release rISG15 hydrogel to facilitate progressive bone regeneration within the tunnel, particularly during the later phases of healing.

The biomechanical assessment further corroborated the influence of rISG15 on graft biomechanical properties (Figure 5B). At 2 weeks, no significant differences in ultimate failure load or stiffness were discernible between the two groups. By week 4, the ultimate failure load within the rISG15-treated group was significantly elevated relative to that of the control group (p < 0.05). At week 8, both the ultimate failure load and stiffness within the rISG15 group were significantly increased compared to the control group (p < 0.01), indicative of enhanced mechanical strength and improved integration at the tendon-bone interface. Conversely, the control group exhibited attenuated progression, with consistently diminished failure load and stiffness values observed across all time points.

Collectively, these findings demonstrate that the sustained-release rISG15 hydrogel effectively potentiates bone tunnel osseointegration and enhances graft biomechanical performance by accelerating bone regeneration and fortifying the structural and mechanical integrity of the tendon-bone interface.

### Sustained-release rISG15 promotes osteogenic differentiation at the tendon-bone interface *in vivo* and *in vitro*

**FIGURE 6:**
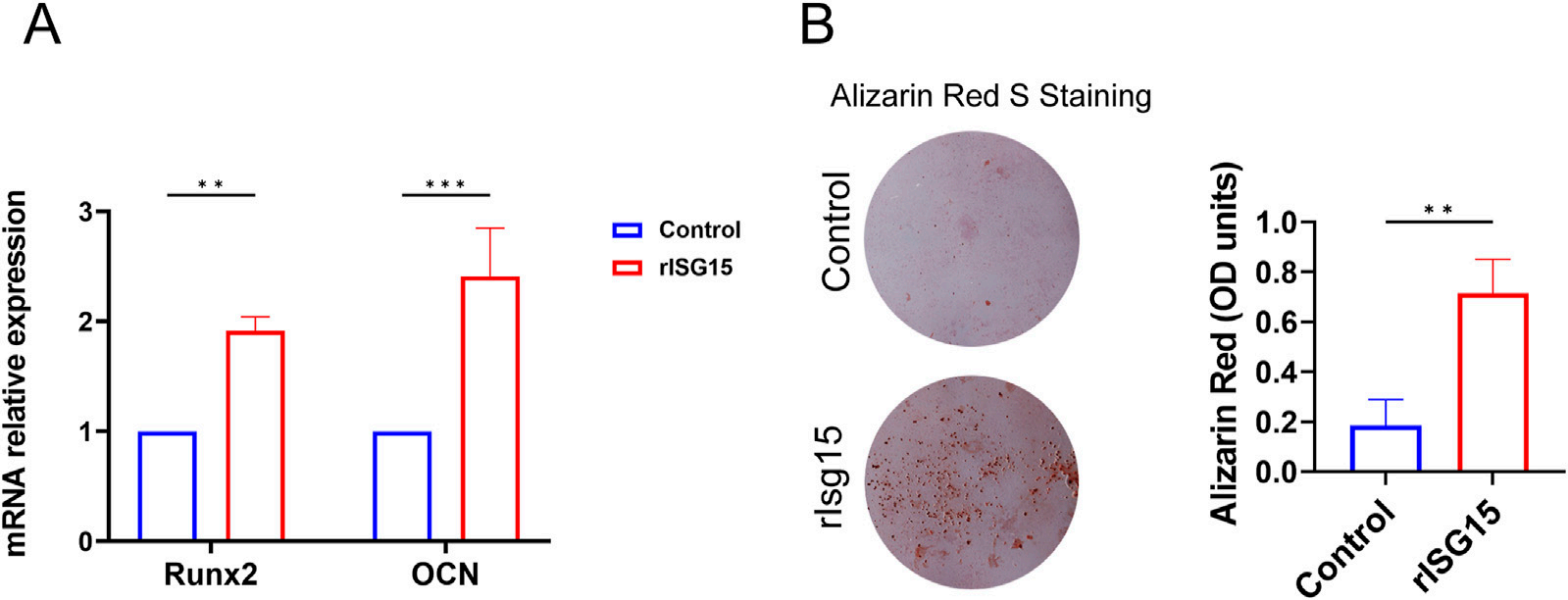
rISG15 Promotes the Osteogenic Differentiation of BMSCs. **(A)** qRT-PCR analysis shows the expression levels of Runx2 and OCN in BMSCs after 14 days of rISG15 stimulation (n = 3 per group). **(B)** Alizarin Red S staining and quantitative analysis demonstrate calcium nodule formation and changes in OD values in BMSCs after 14 days of rISG15 stimulation (n = 3 per group).

To further elucidate the role of sustained-release rISG15 in promoting osteogenic differentiation *in vitro*, we performed an osteogenic differentiation induction assay. BMSCs were cultured in an osteogenic induction medium supplemented with 20 ng/mL recombinant rISG15 for 14 days qRT-PCR analysis revealed a significant upregulation of *Runx2* and *OCN* mRNA expression in rISG15-treated cells compared to the control group (Figure 6A) (*P* < 0.01 for *Runx2*, *P* < 0.001 for *OCN*). Alizarin Red S staining further corroborated enhanced calcium deposition in the rISG15-treated group (Figure 6B). Quantitative analysis demonstrated that optical density (OD) values in the rISG15 group were significantly elevated relative to those in the control group (*P* < 0.01), indicative of increased mineralization. Furthermore, an upregulation of chondrogenic-related genes *(Collagen Ⅱ, Sox9)* mRNA expression was also observed in rISG15-treated cells compared to the control group (Supplementary Figure S2).

**FIGURE 7:**
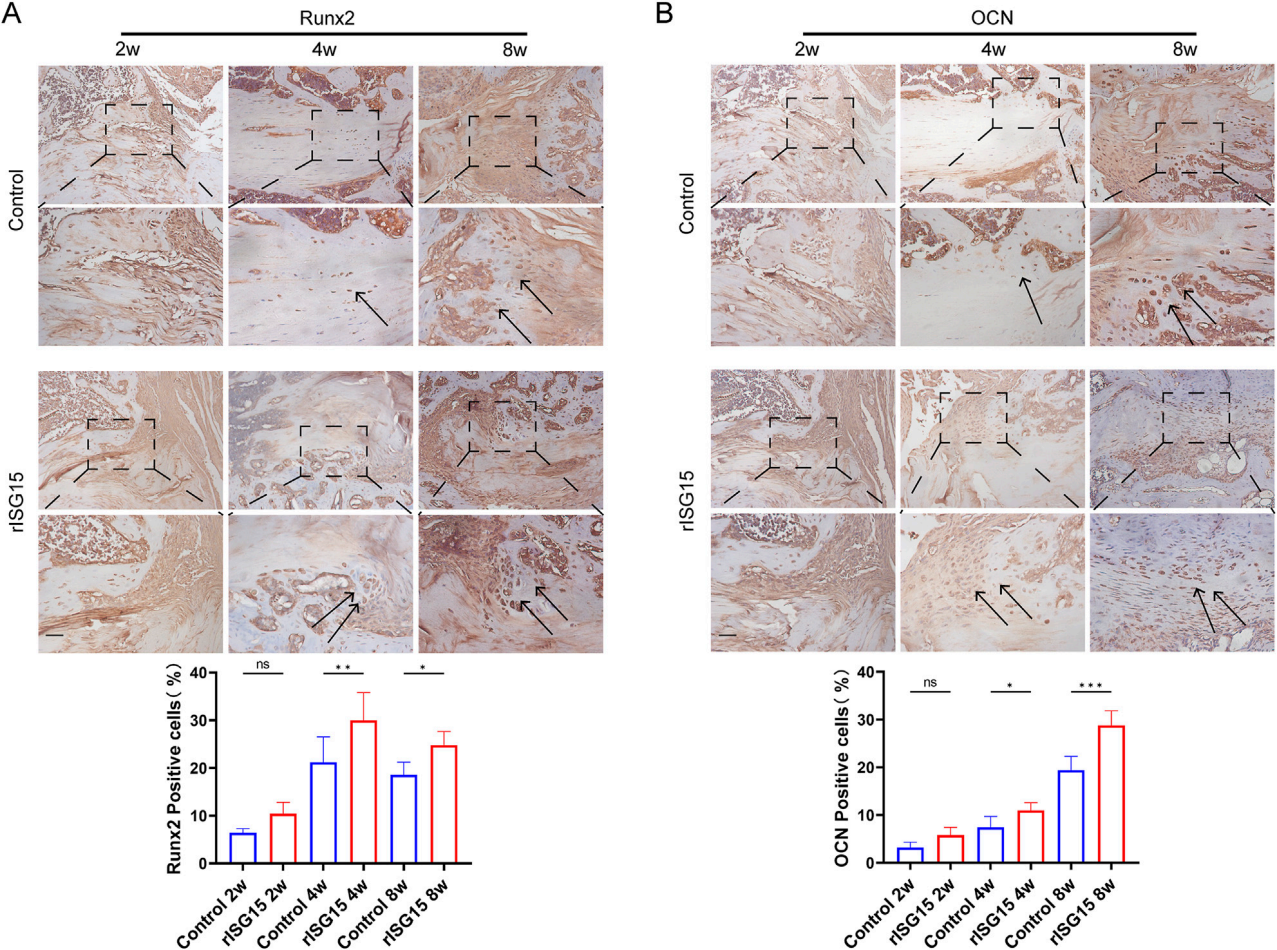
Sustained-release rISG15 enhances osteogenic gene expression at the tendon-bone interface in mice. **(A, B)** Immunohistochemistry and quantitative analysis reveal the expression of Runx2 and OCN at the tendon-bone interface in mice following sustained-release rISG15 treatment at 2, 4, and 8 weeks (n = 5 per group, scale bar = 50 μm).

Immunohistochemical staining assays yielded results consistent with the *in vivo* findings. These analyses demonstrated that *Runx2* and *OCN*, key markers of osteogenic differentiation, were markedly upregulated in the rISG15-treated group compared to the control group at all examined time points (Figures 7A, B). At 2 weeks post-surgery, the distribution of *Runx2*-positive cells in the control group was sparse, whereas the rISG15-treated group exhibited moderate increases. By weeks 4 and 8, the quantity and intensity of *Runx2*-positive cells were significantly greater in the rISG15-treated group, indicative of enhanced early-stage osteogenic differentiation, with statistical significance observed at week 4 (p < 0.01) and week 8 (p < 0.05). Analogously, *OCN* expression, a marker of late-stage osteogenic differentiation, was consistently elevated in the rISG15-treated group, with significant differences noted at week 4 (p < 0.05) and week 8 (p < 0.001) compared to the control group.

## Discussion

To the best of our knowledge, our study represents the first endeavor to investigate the effects of exogenous ISG15 supplementation on tendon bone healing following ACL reconstruction. Our research yielded several salient findings. Initially, we demonstrated that ISG15 enhances the osteogenic differentiation capacity of BMSCs *in vitro*. Secondly, to investigate the regulatory role of ISG15 *in vivo*, we engineered a sustained-release hydrogel encapsulating ISG15 and delivered it into the bone tunnel to counteract the diminished ISG15 secretion resulting from postoperative inflammation. The findings revealed that the rISG15-loaded hydrogel promotes fibrocartilage genesis and collagen deposition over time, thereby facilitating tendon-bone union. Furthermore, the ISG15-treated group exhibited augmented neo-osseous formation, more mature interfacial tissue, and enhanced biomechanical strength at the tendon-bone junction. These findings underscore the propitious role of ISG15 in the ACLR process and suggest that the development of sustained-release delivery systems targeting this cytokine may offer substantial potential for clinical translation.

A paramount determinant of successful ACL reconstruction is the robust integration of the tendon-bone interface. Following ACLR, surgical trauma rapidly precipitates a localized inflammatory cascade, characterized by pronounced infiltration of macrophages and neutrophils into the reconstructed tendon-bone interface. Prior investigations by Rodeo and Rui Geng et al. have demonstrated that absent therapeutic intervention, activated neutrophils, and M1 macrophages predominate at the tendon-bone interface during the early postoperative period, releasing substantial quantities of pro-inflammatory cytokines (Kawamura et al., 2005; Geng et al., 2022). Conversely, M2 macrophages, recognized for secreting cytokines associated with tissue repair, are not observed until day 11 after surgery. A recent study has identified a subset of macrophages expressing CX3CR1+ CCR2+ induced following ACLR (Fujii et al., 2022). These macrophages initiate an early inflammatory program and are subsequently associated with a pronounced interferon (IFN) response. Notably, studies in CCR2-deficient mice have shown increased bone mass, suggesting that CX3CR1+ CCR2+ macrophages may inhibit bone formation at the bone-tendon interface, potentially via their IFN-mediated mechanisms. This evidence underscores that protracted inflammatory cascades can deleteriously affect tissue repair and functional recovery. Consequently, targeting CX3CR1+ CCR2+ macrophages exhibiting active IFN responses may represent a promising therapeutic strategy to enhance outcomes following ACLR.

In the present study, we identified ISG15, a ubiquitin-like protein integral to innate immunity. As a member of the IFN-stimulated gene (ISG) family, ISG15 exists in both a free form and as a covalent modifier of target proteins through enzymatic cascade reactions, underscoring its dual functionality in immune modulation (Takeuchi et al., 2019). While the IFN response is well-established to promote ISG15 secretion, recent studies have demonstrated that ISG15 can stabilize USP18 via non-covalent binding, thereby preventing its ubiquitin-mediated degradation (Zhang et al., 2015). This interaction functions as a regulatory mechanism to temper excessive IFN signaling, protecting tissues from chronic inflammation and consequential damage. Moreover, Chen RH et al. demonstrated that ISG15 stimulation induces macrophages to adopt an M2-like phenotype, typically associated with tissue repair and anti-inflammatory activities (Chen et al., 2020). Furthermore, other studies have linked ISG15 to diverse physiological processes, including inflammation, apoptosis, and autophagy (Zhang et al., 2015). The findings reported by Muhammad et al. further highlight the regulatory role of ISG15 in inflammatory states (Malik et al., 2022). Their study revealed that ISG15-deficient cells exhibit a hyperinflammatory phenotype, characterized by increased synthesis of matrix metalloproteinases (MMPs) and reduced expression of collagens and adhesion molecules. Specifically, silencing ISG15 in fibroblasts resulted in a marked downregulation of collagens (*Col1a1*, *Col5a2*, *Col7a1*, *Col12a1*, *Col14a1*, *Col15a1*) and adhesion molecules (*ITGA11* and *TGM2*), coupled with an upregulation of *MMP1*. These observations suggest that ISG15 may play a pivotal role in maintaining collagen homeostasis and regulating cell migration, processes essential for proper tissue remodeling. In our study, we provided the first *in vitro* and *in vivo* evidence substantiating the role of ISG15 in promoting osteogenic differentiation. These results imply that insufficient ISG15 secretion may lead to impaired bone formation, which could significantly contribute to suboptimal tendon-bone healing.

The native enthesis is a graded region comprising tendon, non-mineralized fibrocartilage, mineralized fibrocartilage, and bone (Voleti et al., 2012). Numerous investigations have sought to replicate this highly specialized transitional interface; however, therapeutic outcomes remain variable. Contemporary research indicates that the biomechanical characteristics of the interface are closely associated with osseous ingrowth and osteointegration, with Sharpey’s fibers serving as an early indicator of successful osteointegration (Hopkinson-Woolley et al., 1994; Anderson et al., 2001; Mihelic et al., 2004). Our micro-CT analysis revealed that both groups exhibited increased microarchitectural parameters between 4 and 8 weeks postoperatively. However, the rISG15 group demonstrated significantly greater neo-osseous formation and bony ingrowth, accompanied by an amelioration in BMD. These findings suggest that ISG15 may attenuate inflammatory bone loss and bolster bone homeostasis, thereby fostering enhanced integration at the tendon-bone interface. The histological assessment further revealed that at 2 weeks postoperatively, both groups presented disorganized fibrovascular tissue infiltrated with inflammatory cells at the interface. However, by 8 weeks the rISG15 group exhibited more prominent Sharpey’s fibers, improved fibril alignment, and a denser interfacial area. These histological alterations were congruent with biomechanical testing results, which demonstrated significantly greater maximum failure load and stiffness in the rISG15 group compared to controls.

Similar to the bone-promoting factor BMP-2, which is steadily advancing towards clinical application, the localized delivery of ISG15 presents several key challenges: (1) rapid local metabolism and a short half-life, thereby limiting its sustained efficacy; (2) uncertain dosage, potentially resulting in complications such as heterotopic ossification; and (3) an initial bolus release, followed by a precipitous decline in concentration below therapeutic thresholds at later stages (Migliorini et al., 2016; Howard et al., 2022). To circumvent these limitations, this study employed a sustained-release hydrogel system for rISG15 delivery, enabling a controlled and prolonged osteoinductive effect through gradual elution. Our results demonstrated that this hydrogel system successfully maintained a stable local ISG15 concentration over a defined period, minimized its degradation, and significantly enhanced reparative outcomes.

Finally, this study is subject to certain limitations. Firstly, we examined a circumscribed number of time points, primarily focusing on the inflammatory and proliferation phases of the healing process, which constrained our capacity to comprehensively assess the effects of ISG15 during subsequent reparative phases. Secondly, this experiment did not explore whether ISG15 directly modulates macrophage polarization. Thirdly, further investigation is warranted to elucidate the precise mechanisms and signaling pathways through which ISG15 facilitates osteogenic differentiation. Moreover, future research should prioritize identifying the optimal ISG15 concentration for enhancing tendon-bone interface healing. Given its established role in modulating autoimmune responses and collagen homeostasis, it is also imperative to engineer stimuli-responsive hydrogels capable of effectively sustaining its therapeutic concentration. Lastly, the findings of this study may have been influenced by potential biases stemming from the limited sample size and inherent variability of the small animal model, which could restrict the generalizability of the results.

## Conclusion

In summation, our findings underscore the multifaceted role of rISG15 in promoting osteogenic differentiation both *in vivo* and *in vitro*. rISG15 facilitates both early and late phases of osteogenesis at the tendon-bone interface. These results suggest that rISG15 may serve as a compelling therapeutic modality for augmenting tendon-bone union in clinical settings.

## Data Availability

The original contributions presented in the study are included in the article/Supplementary Material, further inquiries can be directed to the corresponding author.
