# Supplementary material for: Sustained release of ubiquitin-like protein ISG-15 enhances tendon-to-bone healing following anterior cruciate ligament reconstruction in a mouse model
Source: Front Bioeng Biotechnol. 2025 Mar 12;13:1550584. doi: 10.3389/fbioe.2025.1550584 (PMC11937028; doi:10.3389/fbioe.2025.1550584)
Supplement: Supplementary file 1 [file DataSheet1.docx]

**
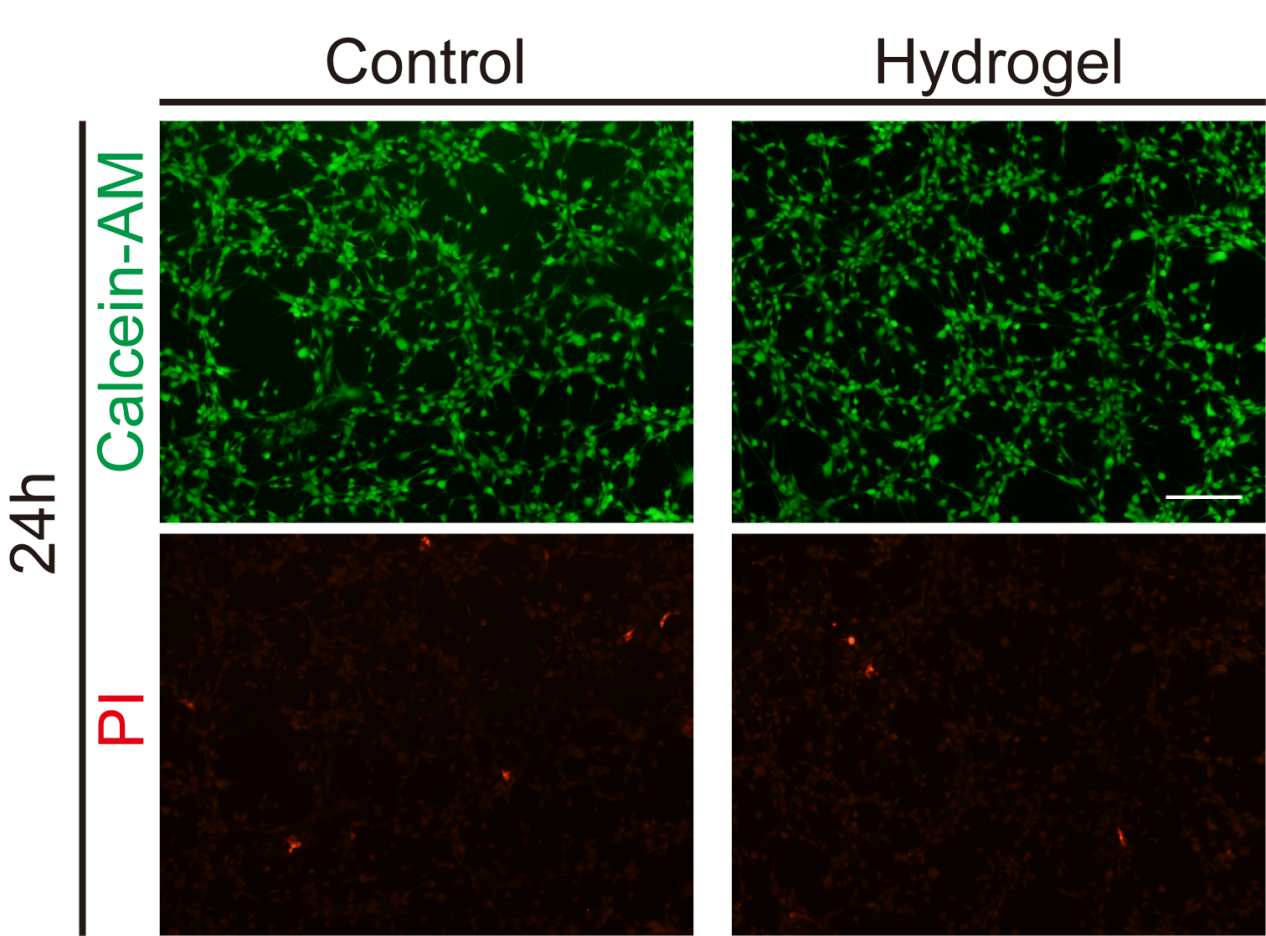
**

**Figure S1. Live/Dead Staining Assay to Assess the Effect of Hydrogel on BMSCs Viability.**

Images of BMSCs co-cultured with hydrogel for 24 hours, stained with calcein-AM (live cells) and propidium iodide (PI, dead cells) (scale bar = 200 μm).


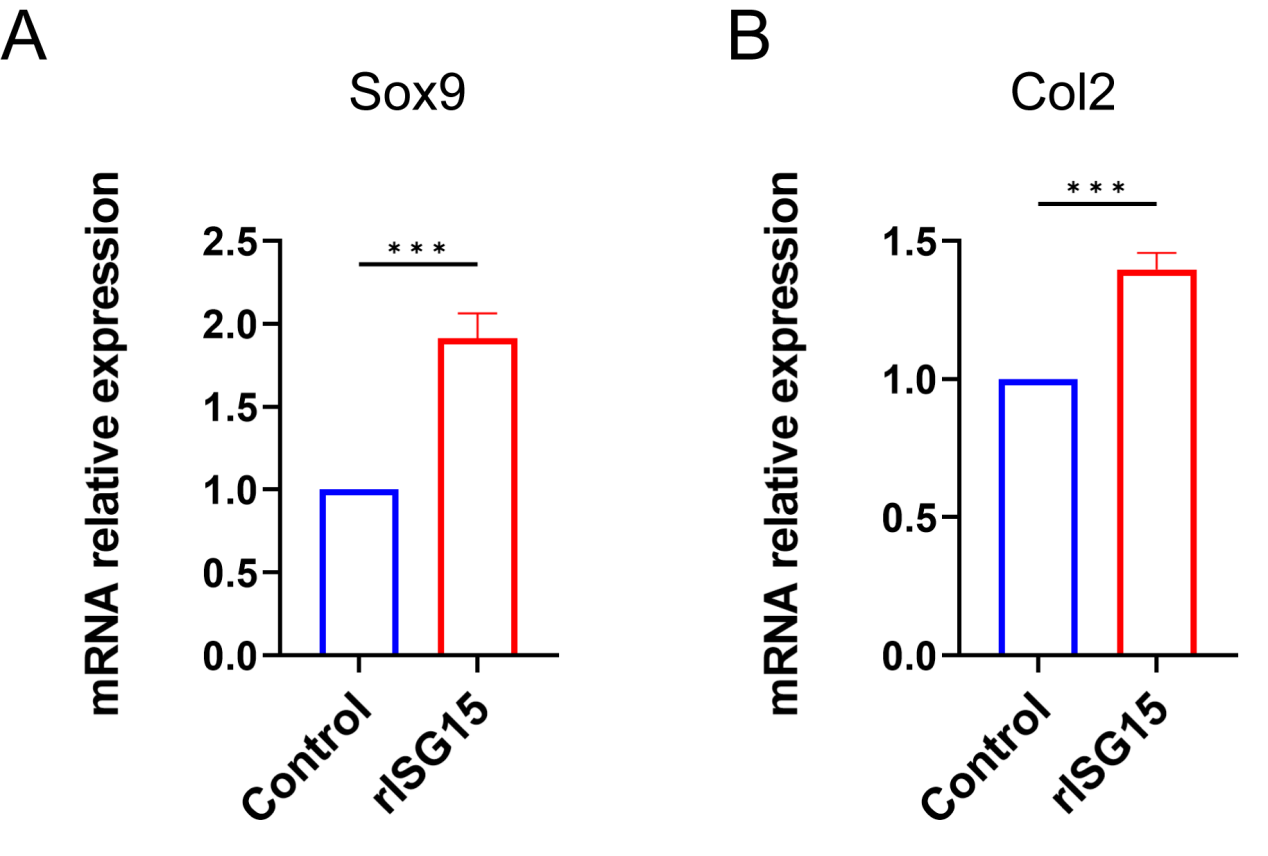


**Figure S2. rIsg15 Promotes Chondrogenic Differentiation of BMSCs.**

A-B. qRT-PCR analysis shows the expressions of *Sox9 and Collagen Ⅱ(Col2)* in BMSCs after 14 days of rISG15 stimulation (n = 3/group).
